# A case control study on the structural equation model of the mechanism of coagulation and fibrinolysis imbalance in chronic schistosomiasis

**DOI:** 10.1097/MD.0000000000006116

**Published:** 2017-02-17

**Authors:** Aiping Le, Lunli Zhang, Wei Liu, Xiaopeng Li, Jianwei Ren, An Ning

**Affiliations:** a Departments of Blood Transfusion; b Department of Infectious Diseases, First Affiliated Hospital of Nanchang University, Nanchang, Jiangxi; c Health Department of the PLA General Staff Headquarters of the Security Council, Beijing; d Jiangxi Institute of Parasitology, Nanchang, Jiangxi, P.R. China.

**Keywords:** chronic schistosomiasis, coagulation and fibrinolysis system, pathogenesis, structural equation model

## Abstract

A structural equation model was used for verification with chronic schistosomiasis to investigate the coagulation–anticoagulation system imbalance and to deduce the mechanism of D-dimer (D-D) level elevation in patients with advanced schistosome hepatic disease. We detected the plasma levels of tissue-type fiber plasminogen activator (tPA), urokinase type plasminogen activator (uPA), plasmin-antiplasmin complex (PAP), plasminogen (PLG), antithrombin (AT), plasminogen activator inhibitor 1 (PAI1), D-D, factor VIII: C (FVIII:C), antithrombin-III (AT-III), PLG, protein S (PS), and protein C (PC) in the healthy people as control (69), patients with chronic schistosomiasis (150) or advanced chronic schistosomiasis (90). FVIII, PAP, D-D, tPA, and uPA plasma levels were significantly higher in the chronic group than in the control group and were also significantly higher in the advanced group. However, AT-III, PC, PS, AT, PLG, and PAI1 plasma levels in the advanced and chronic groups were significantly lower than those in the control group. With progression of disease in patients with schistosomiasis japonica, a hypercoagulable state is induced by the coagulation–anticoagulation imbalance, eventually leading to patients with high levels of D-D. Furthermore, we established a structural equation model path of a “chronic schistosomiasis disease stage–(coagulation–anticoagulation–fibrinolysis)–D-D.” By using analysis of moment structures (AMOS), it was shown that the chronic schistosomiasis stage was positively related to factor VIII and had negative correlation with AT-III; a good positive correlation with PAP, tPA, and uPA; and a good negative correlation with PLG and PAI1. In addition, our results show that the path coefficient of anticoagulation–fibrinolysis system to the chronic stage of schistosomiasis or D-D levels was significantly higher than that of the coagulation system. In conclusion, the coagulation and fibrinolysis imbalance in patients with chronic schistosomiasis, especially with advanced schistosomiasis, is due to the progression of disease stages.

## 1 Introduction

Schistosomiasis is a serious infectious disease caused by *Schistosoma* species and is pathologically characterized as liver inflammation and fibrosis.^[1]^ As one of the major endemic areas, China, especially the southern part, is still facing a threat from *Schistosoma japonicum*, where the number of patients was estimated to be 184,943 and the snail-ridden areas to be 365,468.00 hm^2^ (287.28 hm^2^ of which was newly found).^[2]^ Individuals can be infected with *S japonicum* by contact with contaminated water through which cercariae invade the host organism and develop into schistosomula. Schistosomula then migrate through the circulation system to settle at the portal-mesenteric vein system for final development and maturation. Eventually, the adult *S japonicum* mate and lay eggs, which are transferred through blood flow and deposited in the liver. Inside the eggs, myracidium induce a local inflammation response including egg granuloma and liver fibrosis, and this eventually leads to the chronic infectious status of the host.^[1]^ Infected people with repeated infections of mass cercariae in contaminated water or without proper treatment may develop to the late stage of the disease with more severe symptoms including hepatosplenomegaly, liver portal hypertension, and complications such as hypersplenism and esophageal variceal rupture bleeding.^[3]^

The life cycle of adult *S japonicum* including its parasitism and migration in the vein system and deposition of eggs in the liver tissue causes specific pathologic responses in the host.^[4]^ Theoretically, blood vessel injuries first induce a local inflammatory response and subsequent imbalance of coagulation and fibrinolysis, each of which may interact with the other and eventually lead to the amplified and systemic pathologic response of the host. Systemic coagulation plays an important role in the compensation of parasite immunity as the subsequent response of inflammation against the parasitism of *S japonicum*.^[5–7]^ However, to maintain the homeostasis of the blood system, over-secreted fibrins in coagulation need to be further degraded through fibrinolysis factors such as plasminogen and plasmin.^[8]^ Previous studies reported abnormal blood coagulation status in patients with schistosomiasis japonica,^[5–7]^ and our former studies also found increased levels of D-D, especially in patients with advanced schistosomiasis.^[8]^ However, few studies comprehensively examined the coagulation and fibrinolysis system together in patients with schistosomiasis japonica. The mechanism of the imbalance of the coagulation and anticoagulation system and the abnormality of the fibrinolysis system in patients with chronic schistosomiasis (especially advanced schistosomiasis) remain unclear. Here, in the endemic area of *S japonicum* in China, we recruited patients both with chronic and advanced schistosomiasis as well as healthy controls to investigate the variation in coagulation and anticoagulation, and fibrinolysis and antifibrinolysis levels at different disease stages in chronic schistosomiasis patients and further verified the occurrence and its mechanism of coagulation and fibrinolysis imbalance in patients with chronic schistosomiasis.

## 2 Materials and methods

### 2.1 Study design and participants

Patients infected with schistosomiasis, previously diagnosed, treated, and recorded in the *Jiangxi Information Management System for Schistosomiasis Treatment and Assistance* were randomly selected in Xinjian, Nanchang, Duchang, and Yugan of Jiangxi province, China. Patients with viral hepatitis and tuberculosis were excluded. A total of 240 patients with schistosomiasis japonica, consisting of 150 patients with chronic disease (chronic group) and 90 patients with advanced disease (advanced group), were finally recruited. The control group comprised 69 healthy individuals who visited the First Affiliated Hospital of Nanchang University for routine medical examination. This study was approved by the Human Research Ethics Committee of the First Affiliated Hospital of Nanchang University. All patients received an explanation about the scope of the study, such as objectives, procedures, and potential risks, and signed an informed consent statement before inclusion in the study.

### 2.2 Parasitological diagnosis

Stool samples from the control and patient groups were taken on 2 consecutive days, and each sample was tested twice by the Kato–Katz method. The mean egg counts were reported. Information on physical examination and history of clinical diagnostics and treatment were accessed from the *Jiangxi Information Management System for Schistosomiasis Treatment and Assistance*. On the basis of the comprehensive information of the history of clinical diagnosis and treatment, physical examination, and abdominal ultrasonography, the disease stage of the patients with schistosomiasis japonica was determined strictly in accordance with the diagnostic criteria for schistosomiasis.^[9]^

### 2.3 Blood sample collection and processing

Venous blood samples, in the morning and after fasting, of all the participants were collected under aseptic conditions in vacuum tubes containing 0.562 M EDTA-K2 and promoting coagulating tubes (Lucky Nation Medical, Youzhou, China); the sample capacity was 2 and 3 mL, respectively. Blood plasma and serum were obtained after rapid centrifugation for 10 minutes at 2000×*g* and stored in 0.5 mL aliquots at −80°C.

### 2.4 Blood sample tests

The routine blood test including white blood cell count (WBC), red blood cell count (RBC), hemoglobin (Hb), platelet (PLT), and leukocyte differential count was analyzed by Hematology Analyzer (sysmexXE-2100, Japan). Liver function tests included aspartate and alanine aminotransferases (AST and ALT), total bilirubin (TB), direct bilirubin (DB), total protein (TP), albumin protein (ALB), globulin protein (GLB), albumin/globulin ratio (A/G), glutamyltranspeptidase (GGT), and alkaline phosphatase (ALP) and were analyzed by automated biochemistry analyzer (Hitachi-7600, Japan). Liver fibrosis tests included hyaluronic acid (HA), type III procollagen (PCIII), type IV collagen (IV-C), and laminin (LN) and were also analyzed by the chemiluminescence method (MAGLUMI4000, China). Blood coagulation and fibrinolysis tests were performed using a full-automatic coagulation analyzer (sysmex CA-8000, Japan) for prothrombin time; thrombin time (TT); activated partial thromboplastin time (APTT); fibrinogen (FBG); D-D; and the activities of blood coagulation factor VIII and AT-III, PLG, PS, and PC. All the reagents used were those specified for the instrument. Enzyme-linked immunosorbent assay (ELISA, UscnLifeScience, China) was used to detect the level of plasma tPA, urokinase type plasminogen activator (uPA), PAP, PLG, AT, and PAI1. All tests were carried out strictly in accordance with the standard operating procedures (SOPs).

### 2.5 Data analysis

#### 2.5.1 Statistical analysis

Test data of normal distribution or near normal distribution are expressed as mean and standard deviation (SD), and test data of skewness distribution are expressed as median (interquartile range, IQR). Comparisons between groups for gender were performed using the *χ*^2^ test. Student *t* test and 1-way ANOVA were used for within-group comparisons for continuous variables. The Mann–Whitney *U* test was used for nonparametric comparisons. All the above analyses were performed using SPSS version 19.0 for Windows (IBM, USA). All reported *P* values were 2-sided, and *P* values <0.05 were considered statistically significant.

#### 2.5.2 Verification analysis of structural equation model

The theoretical model was established according to the variation tendency of the level of coagulation and anticoagulation and fibrinolysis and antifibrinolysis in different disease stages of chronic schistosomiasis. We speculated that the disease stage of chronic schistosomiasis would directly affect the coagulation system, anticoagulation system, fibrinolysis system, and levels of D-D. The coagulation, anticoagulation, and fibrinolysis systems directly affect the levels of D-D for constructing the initial path diagram and the specific measurement indicators. Accordingly, the chronic schistosomiasis disease stage was defined as the observed variable, and according to the staging score, the control group was designated as 1, the chronic group as 2, and the advanced group as 3, representing the severity of the disease. The coagulation system was also defined as the observed variable, and the observation indicator was factor VIII, which represents the level of blood coagulation. The coagulation and fibrinolysis system was defined as the latent variable, and the observed indicators were tPA, uPA, PAL1, PAP, PLG, and ATIII, which represented the fibrinolysis and anticoagulation levels, respectively. D-D was defined as the observed variable, and the observation indicator was the D-D level. The defined variables were used for descriptive analysis and correlation analysis, and the limiting correlation coefficient was −1 to 1, which determined whether there was a correlation. The path coefficients were estimated using the maximum likelihood method, and the path diagram with the path coefficient (pc) was obtained. A positive number indicated positive correlation, while a negative number indicated negative correlation; the greater of the absolute values showed greater influence on the upper indicators, and vice versa. We used the *χ*^2^test and standard fitting degree and significance test of the pc of the structural equation model to evaluate the quality of the model and the fitting degree of the model to the data. Finally, we determined whether the causal relationship between the parameters and the default model was reasonable. All the above analyses were performed using AMOS version 22.0 for Windows (IBM, USA). *P* values of the *χ*^2^ test of the structural equation model greater than 0.05 were not considered statistically significant. *P* values of the pc significance test of less than 0.01 were considered statistically significant.

## 3 Results

### 3.1 Comparisons of routine blood test, liver function, and liver fibrosis

As shown in Table 1, with the progression of the disease stage of schistosomiasis japonica, the indicators of liver function in patients, for example, ALT, AST, TB, DB, GGT, GLB, ALP, and of liver fibrosis, for example, HA, PCIII, IV-C, and LN, showed an increasing trend; this was more serious in advanced schistosomiasis. However, the number of white cells, red cells, and platelets showed a decline, which was also more obvious in advanced schistosomiasis. There was a significant difference among the chronic group, advanced group, and control group (*P* *<* 0.05).

**Table 1:**
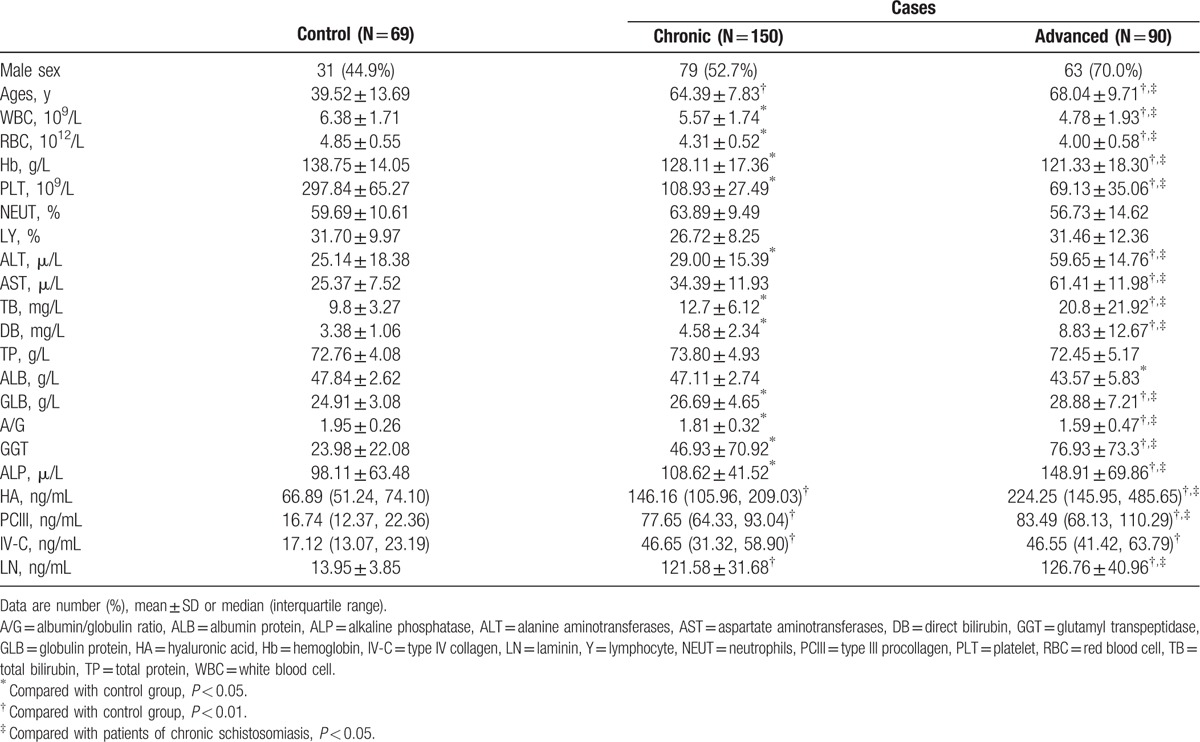
Comparisons of routine blood tests and liver function in the schistosomiasis and control groups.

### 3.2 Variation trend of coagulation and anticoagulation and fibrinolysis and antifibrinolysis factors

With the progression of the stage of disease of schistosomiasis japonica, coagulation duration showed no significant difference in chronic schistosomiasis (*P* > 0.05). But FVIII:C followed an increasing trend and the level of activity of AT-III, PC, and PS decreased in the control group, chronic group, and advanced group successively, with statistically significant differences among the groups (*P* < 0.05). The levels of D-D, tPA, uPA, and PAP showed an increasing trend in the control group, chronic group, and advanced group, while the PLG and PAI1 levels showed a downward trend; there were statistically significant differences among the groups (*P* < 0.05), as shown in Table 2.

**Table 2:**
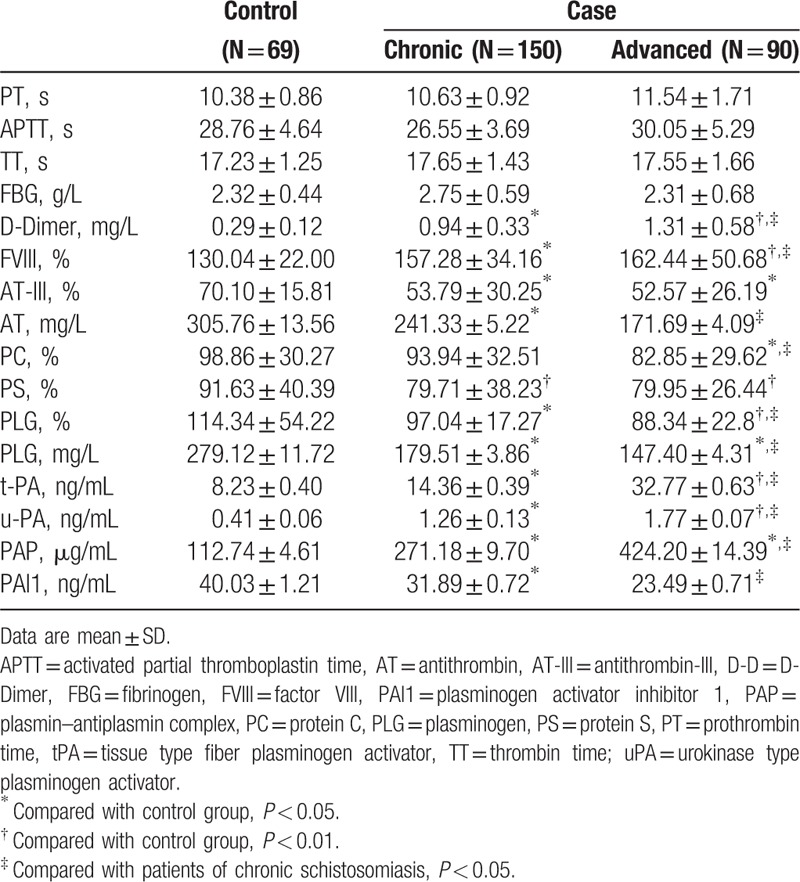
Comparisons of factors of coagulation and fibrinolysis in the schistosomiasis and control groups.

### 3.3 Validation and analysis of the structural equation model for the imbalance of coagulation and fibrinolysis system in chronic schistosomiasis

#### 3.3.1 Establishment of theoretical model and initial path graph

**Figure 1:**
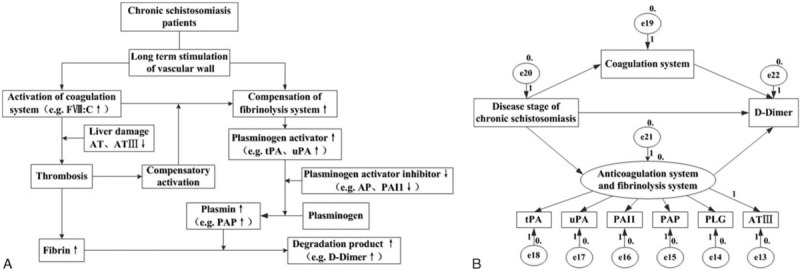
A, The speculative path graph of the theoretical model of the imbalance of the coagulation and fibrinolysis system in chronic schistosomiasis. B, The initial path diagram of the structural equation model of the imbalance of coagulation and fibrinolysis system in chronic schistosomiasis.

According to variation of the coagulation, anticoagulation, fibrinolysis, and antifibrinolysis factor levels in the different stages of chronic schistosomiasis, we established the theoretical model of the imbalance of coagulation and fibrinolysis systems in chronic schistosomiasis. With the progression of disease stages, patients with chronic schistosomiasis developed a hypercoagulable state induced by coagulation and anticoagulation imbalance, thereby causing compensatory hyperfibrinolysis, and eventually leading to the production of high levels of D-D in patients with advanced disease. As shown in Fig. 1A and B, an initial path diagram of the structural equation model was established using AMOS software, focusing on the study of “chronic schistosomiasis staging-coagulation system-D-D,” “chronic schistosomiasis staging-anticoagulation and fibrinolysis system-D-D,” and “chronic schistosomiasis staging-D-D.” Descriptive statistics of the original data are shown in Table 3.

**Table 3:**
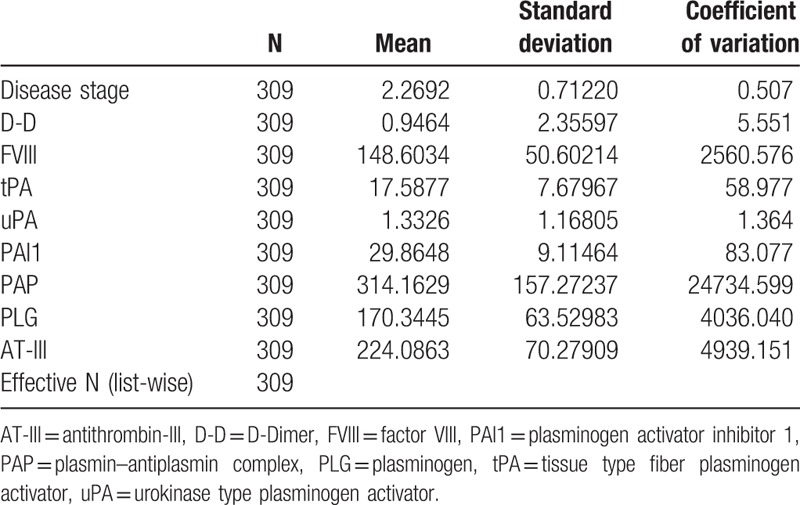
Descriptive statistics of the original data of chronic schistosomiasis.

#### 3.3.2 Correlation analysis

Correlation analysis of each observation indicator of chronic schistosomiasis showed a high correlation, which indicated that the observation indicator can be further used to construct the structural equation model, as shown in Table 4.

**Table 4:**
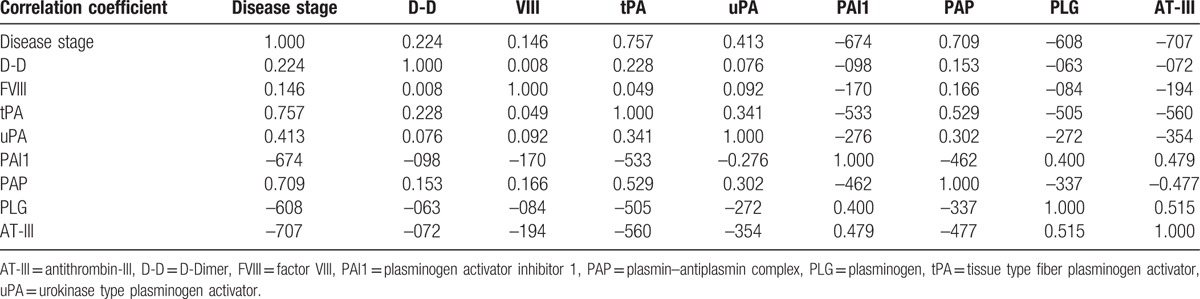
Correlation matrix of the observation indicators of chronic schistosomiasis.

#### 3.3.3 Verification analysis of the structural equation model

**Figure 2:**
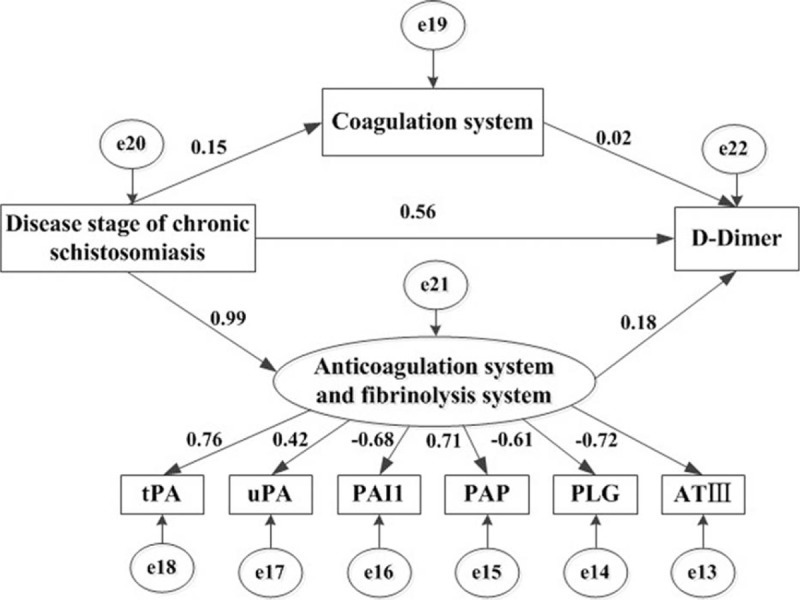
The path diagram of the structural equation model of the imbalance of coagulation and anticoagulation and fibrinolysis system in chronic schistosomiasis.

We carried out the default model analysis of the abovementioned parameters in Fig. 1 B by AMOS to test whether the data matched the structural equation model path and obtained the path coefficients of the structural equation model path diagram fitting by the maximum likelihood method (Fig. 2). As shown in the analysis of the results of the pc of the structural equation model, there was a good positive correlation of chronic schistosomiasis disease stage with factor VIII, but a negative correlation with AT-III, and the path coefficients were 0.15 and −0.72 respectively. In addition, a positive correlation was found between the chronic schistosomiasis disease stage and levels of PAP, TPA, uPA, and their path coefficients were 0.71, 0.76, and 0.42 respectively, but a negative correlation was found with levels of PLG and PAI1, and their path coefficients were −0.61 and −0.68. The pc of the anticoagulation and fibrinolysis system (pc = 0.99) was significantly higher than the D-D level (pc = 0.56) to the disease stage of chronic schistosomiasis. Moreover, the pc of the anticoagulation and fibrinolysis system to the D-D levels is 0.18, which was higher than the coagulation system (pc = 0.02).

#### 3.3.4 Test of the structural equation model

Verification analysis of the structural equation model proves that the causal relationship of the theoretical model is well validated. The *χ*^2^ test for the structural equation model had a significant probability greater than 0.05 (*χ*^2^=28.768, *P* = 0.274), which indicated that the model was fit for data and convergence. Parameter estimation showed that the path coefficients had reached a significant level (*P* < 0.001), which indicated that the estimated parameters were in a reasonable range (as shown in Table 5). Fitting degree analysis showed that the normed fit index (NFI) was more than 0.9 (NFI = 0.957), which showed that the model fit was high.

**Table 5:**
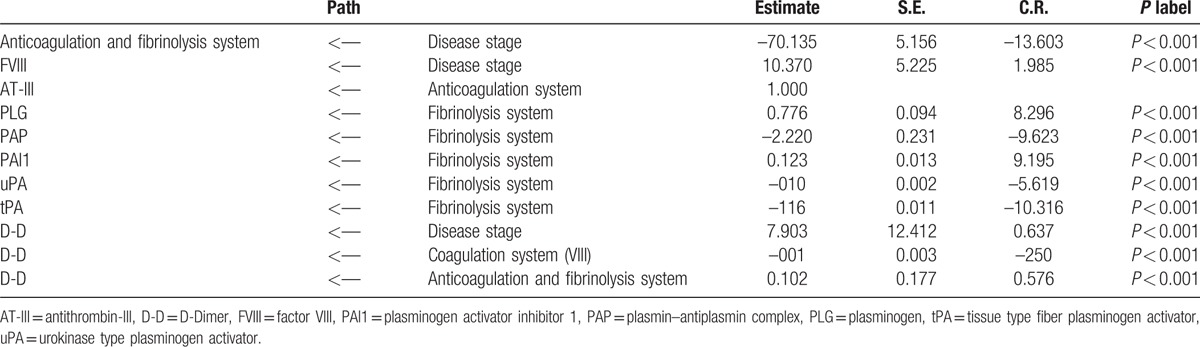
Parameter estimation of the structural equation model.

## 4 Discussion

The existence of an abnormal coagulation state has been reported in chronic schistosomiasis patients, especially with advanced schistosomiasis. Our previous study also found that the D-D levels of the patients with chronic schistosomiasis were significantly higher than that of healthy people, and were especially evident in patients with advanced schistosomiasis.^[8]^ If there is a balance between the coagulation and anticoagulation systems and fibrinolysis and antifibrinolysis systems, it is possible to have a hypercoagulable state or a bleeding tendency. The increase in FVIII:C was mainly seen in a hypercoagulable state and thrombotic disease,^[10–12]^ and the lack of AT-III and AT may lead to thrombosis.^[13,14]^ PC and PS activity increased to inhibit the coagulation process by inactivating VIIIα and Vα.^[15,16]^ Plasminogen will be activated to produce plasmin by tPA and uPA, which play an important role in physiological hemostasis and thrombus degradation.^[17,18]^ The increase in tPA and uPA activity and decrease in PAI1 activity led to the decrease in plasminogen, the production of plasmin, and the decrease in antiplasmin level. There was an increase in D-D, which resulted in the body showing hyperfibrinolysis.^[19]^

Analysis of the results showed an increasing trend for the levels of coagulation factors and a decreasing trend for anticoagulation factors with no significant difference in coagulation duration with progression of disease stage in patients with chronic schistosomiasis (*P* < 0.05); this resulted in a hypercoagulable state of the coagulation and anticoagulation systems. This condition was conducive to the formation of thrombus and was not related to the number of platelets. The main reason for this condition was related to the pathological basis of chronic schistosomiasis. A large number of eggs were deposited in the mesenteric vessels after schistosome infection^[19]^; the eggs and antigens stimulated the vascular wall and activated the coagulation system. However, with the progression of disease stages, patients with chronic schistosomiasis developed a hypercoagulable state induced by the lack of anticoagulation activity, especially in advanced schistosomiasis patients with liver damage, which indicated worsening liver function associated with the development of disease. The hypercoagulable state of the body promoted the activation of fibrinolysis system and inhibition of antifibrinolysis activity, eventually leading to high levels of D-D. In this progression, the activation of fibrinolysis system was dominant, and the antifibrinolysis activity was obviously insufficient, which may be related to the intravascular thrombosis in the patients with chronic schistosomiasis.

Factor analysis can reflect the relationship of variables, but cannot analyze the causal relationship of variables. Path analysis can analyze the causal relationship of variables, but its basic assumptions are difficult to apply in an actual situation. The structural equation model integrates path analysis, confirmatory factor analysis, and general statistical test methods, which can analyze the causal relationship among multiple sets of variables. This model can not only determine the relationship between observable variables and unobservable latent variables, but it can also study the direct effect, indirect effects, and effect magnitude and direction of latent variables, which cannot be solved by the traditional statistical methods.^[20]^ The structural equation model can be used for confirmatory analysis of multivariate variables and verify whether the causal relationship of variables exists.

In this study, the variation tendency of coagulation and anticoagulation, and fibrinolysis and antifibrinolysis factors was detected and analyzed in different stages of patients with chronic schistosomiasis. The results showed that the hypercoagulable state induced by the coagulation–anticoagulation imbalance caused compensatory hyperfibrinolysis eventually leading to the production of high levels of D-D in patients, which increased with the progression of the disease stage of patients with schistosomiasis. On the basis of this finding, we hypothesized and established a theoretical path model of the structural equation model for chronic schistosomiasis stage-(coagulation–anticoagulation– fibrinolysis)-D-D. According to the analysis of the default model for the primary data of coagulation, anticoagulation, fibrinolysis, and antifibrinolysis in the different stages of patients with chronic schistosomiasis, and the pc estimation by using the maximum likelihood method, the results showed that the disease stage of chronic schistosomiasis and the level of parameters had good correlation. In addition, the *χ*^2^ test (*χ*^2^=28.768, *P* = 0.274), fitting degree analysis (NFI=0.957), and pc significance test (*P* < 0.001) proved that the model had a high degree of convergence, good fit, and reasonable parameters. This showed that the causal relationship of the theoretical model path was reasonable, and that the theoretical model had been well verified in the verification analysis of the structural equation model. The path analysis results of the structural equation model showed that the disease stage of chronic schistosomiasis had a positive correlation to the level of coagulation and was negatively correlated with the level of anticoagulation, and the pc of anticoagulation was dominant. This indicated that in the chronic schistosomiasis patients with progression of disease stages, the coagulation level was upregulated to a certain extent and the anticoagulation level was downregulated to a certain extent and was dominant. This leads to the development of a hypercoagulable state in the body, which is induced by the coagulation–anticoagulation imbalance. At the same time, the disease stage of chronic schistosomiasis had a positive correlation with the level of fibrinolysis and was negatively correlated with the level of antifibrinolysis. This showed that the level of fibrinolysis with the progression of the disease stages in patients with chronic schistosomiasis was increased to a certain extent, and the level of antifibrinolysis was reduced, resulting in the manifestations of abnormal hyperfibrinolysis. The pc of the anticoagulation and fibrinolysis system to the chronic schistosomiasis disease stage or D-D levels was significantly higher than that of the coagulation system, indicating that the anticoagulation and fibrinolysis systems accounted for the main advantage in the progression of coagulation and fibrinolysis imbalance and D-D upregulation. Our results also suggest that the anticoagulation and fibrinolysis status may reflect the staging of chronic schistosomiasis to a certain extent. In the previous study, the portal blood flow resistance might cause terrible thrombosis and collateral circulation in schistosomiasis, known as cavernous transformation.^[21]^ Although the relationship of staging of chronic schistosomiasis and the anticoagulation and fibrinolysis status was recovered in this study, there was still some limitations, for example the lack of age-matched control, replication validation of the model, and the potential bias or imprecision of the study.

In summary, we found that coagulation and fibrinolysis imbalance in patients with chronic schistosomiasis, especially advanced schistosomiasis, was due to the progression of disease stages and coagulation and anticoagulation imbalance caused by the hypercoagulable state and high levels of D-D by using the structural equation model analysis and verification. It was an important clinical significance in clinical treatment of coagulation and fibrinolysis balance in patients with chronic schistosomiasis, especially with advanced schistosomiasis. However, the indicator number of coagulation and fibrinolysis is still limited in the study, and we focused on improving the number of indicators, verified and analyzed more complex paths on the basis of the theoretical model by using confirmatory analysis of the structural equation model.
